# Role of long non-coding RNA-RNCR3 in atherosclerosis-related vascular dysfunction

**DOI:** 10.1038/cddis.2016.145

**Published:** 2016-06-02

**Authors:** K Shan, Q Jiang, X -Q Wang, Y -N -Z Wang, H Yang, M -D Yao, C Liu, X -M Li, J Yao, B Liu, Y -Y Zhang, Yong J, B Yan

**Affiliations:** 1 Eye Hospital, Nanjing Medical University, Nanjing, China; 2 The Fourth School of Clinical Medicine, Nanjing Medical University, Nanjing, China; 3 Department of Cardiology, Shanghai Tenth People's Hospital, Tongji University School of Medicine, Shanghai, China; 4 Department of Cardiac Surgery, The First School of Clinical Medicine, Nanjing Medical University, Nanjing, China; 5 Key Laboratory of Cardiovascular Disease and Molecular Intervention, Nanjing Medical University, Nanjing, China

## Abstract

Atherosclerosis is one of the most common vascular disorders. Endothelial cell (EC) dysfunction and vascular smooth muscle cell (VSMC) proliferation contributes to the development of atherosclerosis. Long non-coding RNAs (lncRNAs) have been implicated in several biological processes and human diseases. Here we show that lncRNA-RNCR3 is expressed in ECs and VSMCs. RNCR3 expression is significantly upregulated in mouse and human aortic atherosclerotic lesions, and cultured ECs and VSMCs upon ox-LDL treatment *in vitro*. RNCR3 knockdown accelerates the development of atherosclerosis, aggravates hypercholesterolemia and inflammatory factor releases, and decreases EC and VSMC proliferation *in vivo*. RNCR3 knockdown also reduces the proliferation and migration, and accelerates apoptosis development of EC and VSMC *in vitro.* RNCR3 acts as a ceRNA, and forms a feedback loop with Kruppel-like factor 2 and miR-185-5p to regulate cell function. This study reveals that RNCR3 has an atheroprotective role in atherosclerosis, and its intervention is a promising strategy for treating atherosclerosis-related vascular dysfunction.

Atherosclerosis is one of the most common vascular disorders, which is the underlying cause of clinical manifestation of myocardial infarction, stroke, and gangrene.^1, 2^ Atherosclerosis is usually characterized by endothelial damage, inflammatory cell, and vascular smooth muscle cell (VSMC) accumulation, as well as extracellular lipid and fibrous tissue deposition. Endothelial dysfunction is considered as an early marker for atherosclerosis, preceding angiographic or ultrasonic evidence of atherosclerotic plaque. Generally, endothelial cells (ECs) are highly adaptive to environmental cues, such as hyperlipidemia and inflammation stimulus. Damaged ECs are rapidly replaced by the proliferation of resident ECs. However, persistent injury could induce EC injury and apoptosis in atherosclerosis.^3, 4^ Atherosclerosis develops preferentially at the site where disturbed laminar flow compromises EC function, and then are followed by chronic inflammatory response and VSMC proliferation. VSMCs are activated and regain their highly proliferative characteristics at certain pathological condition, thus contributing to the thickening and stiffening of arterial wall. Activated VSMCs have an important role in the progression and eventual rupture of atherosclerotic plaques.^5, 6, 7^ Thus, strategies to prevent EC and VSMC dysfunction may provide novel therapeutic approach to reduce atherosclerosis-related vascular diseases.

Long non-coding RNAs (lncRNAs) constitute a class of transcripts longer than 200 nucleotides.^8^ They regulate gene expression at epigenetic, transcription, and translation levels, coordinating and integrating multiple signaling pathways involved in cellular differentiation, proliferation, homeostasis, and organ development.^9, 10^ Several lines of evidence have shown that lncRNAs are pivotal regulators of EC and VSMC proliferation, differentiation, and cell motility.^11, 12, 13, 14^ Atherosclerosis is characterized by abnormal proliferation, migration, and pro-inflammatory activation of ECs and VSMCs. Thus, we speculated that lncRNAs are potential regulators of atherogenesis.

Retinal non-coding RNA3 (RNCR3), also known as LINC00599, is a long intergenic non-protein coding RNA, which was first reported to be dynamically expressed during mouse retinal development.^15^ RNCR3 is shown as a regulator of neurons and oligodendrocyte differentiation.^16^ RNCR3^−/−^ mice exhibit neuronal dysfunction and aberrant growth of dentate granule cell axon.^17^ Although functionally different, nervous and vascular systems usually share common regulators for function maintenance.^18^ Moreover, our preliminary experiments reveal that RNCR3 knockdown affects ocular microvascular dysfunction. The vasculature of the eye and the heart share many common characteristics.^19^ In this study, we investigated the role of RNCR3 in atherogenesis. Our studies indicate that RNCR3 expression is significantly increased in mouse and human aorta atherosclerotic lesions. RNCR3 exerts a remarkable atheroprotective effect on atherogenesis via RNCR3/Kruppel-like factor 2 (KLF2)/miR-185-5p regulatory network. LncRNA-RNCR3 is a promising target for treating atherosclerosis and related cardiovascular disorders.

## Results

### LncRNA-RNCR3 is involved in vascular dysfunction: evidence from ocular study

**Figure 1:**
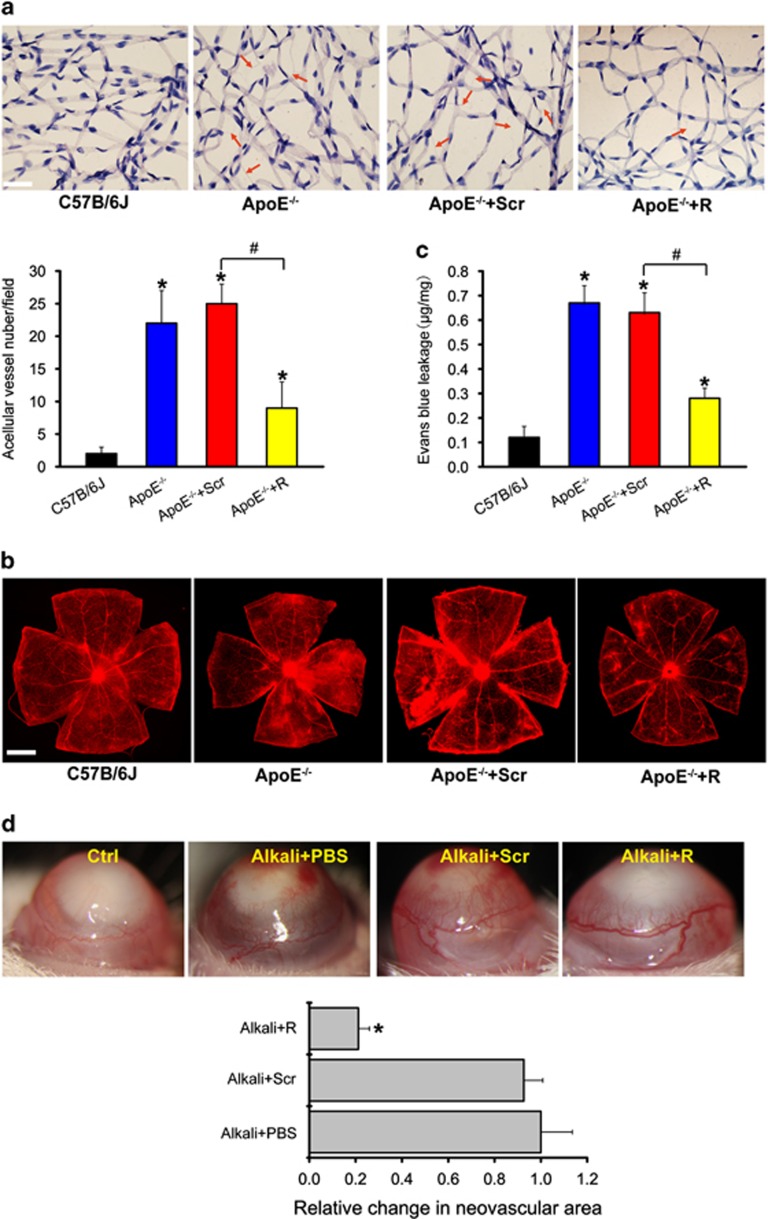
LncRNA-RNCR3 is involved in ocular vascular dysfunction. (**a**–**c**) Four-week-old male ApoE^−/−^ mice were fed with high-fat diet containing 0.15% cholesterol and 20% fat for 16 weeks. They were received subcutaneous injection of scrambled shRNA (ApoE^−/−^+Scr) or RNCR3 shRNA viral vector (ApoE^−/−^+R), or left untreated (ApoE^−/−^). shRNA injection was started at 4 weeks after feeding with high-fat diet. Viral vector was injected once every 2 weeks. Age-matched wild-type C57B/6J mice were used as the control group (C57B/6J). Retinal trypsin digestion was performed to detect the change of acellular capillaries. Red arrows indicated acellular capillaries. Acellular capillaries were quantified in 20 random fields per retina and averaged (A, *n*=4, scale bar, 20 *μ*m). The above-mentioned groups were infused with Evans blue dye for 2 h. The fluorescence signaling of flat-mounted retina was observed using a microscope. A representative image was shown. Scale bar, 100 *μ*m (**b**). Quantification of Evans blue leakage was conducted (**c**, *n*=4). *P<0.05 *versus* C57B/6J group; ^#^*P*<0.05 ApoE^−/−^+R *versus* ApoE^−/−^+Scr group. (**d**) Four-month-old male C57B/6J mice were received alkali burn on the central corneas or left untreated (Ctrl), and then received an injection of RNCR3 shRNA (R), scrambled shRNA (Scr), or PBS. Four days after alkali burn, corneal neovasculature was observed by slit-lamp and vascular area was quantified (*n*=4). *P <0.05 *versus* Alkali+PBS group

There is interplay between cardiovascular functions and risk factors, and the occurrence and progression of many ocular diseases.^19^ We first employed the eye to investigate the role of RNCR3 in vascular dysfunction due to the easily accessible vessels. Atherosclerosis resulted in the occurrence of new blood vessels in retinas, while RNCR3 knockdown obviously attenuated retinal neovascularization (Figure 1a). Atherosclerosis led to serious retinal capillary leakage. By contrast, RNCR3 knockdown obviously alleviated capillary degeneration and capillary leakage in ApoE^−/−^ mice (Figures 1b and c). We further employed corneal neovascularization model to investigate the role of RNCR3 in angiogenesis. Vessels started to grow from the limbal arcade toward alkali burn site. RNCR3 knockdown could significantly reduce alkali burn-induced corneal neovascularization in C57BL/6J mice (Figure 1d). Collectively, these evidences from ocular studies show that RNCR3 is involved in vascular dysfunction.

### LncRNA-RNCR3 is upregulated in aortic atherosclerotic lesions and its knockdown aggravates atherosclerosis *in vivo*

**Figure 2:**
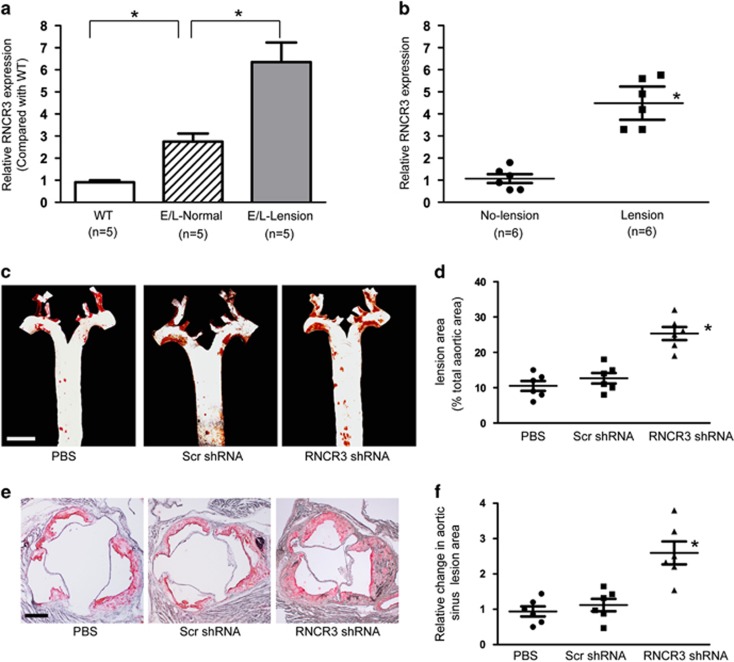
LncRNA-RNCR3 is upregulated in aortic atherosclerotic lesion and its knockdown aggravates atherosclerosis *in vivo*. (**a**) RNCR3 expression in the aorta of 5-month-old male ApoE^−/−^ and C57B/6J mice was determined by qRT-PCRs and normalized to the expression of GAPDH. The data were expressed as relative mRNA expression compared with average expression in wild-type group (WT). WT, wild type C57B/6J mice. E/L-lesion and E/L-normal: aorta segments with atherosclerotic lesions or without lesion (normal) from apoE^−/−^ mice, respectively (*P<0.05). (**b**) RNCR3 expression was detected in atherosclerotic lesions and non-lesional aortic intimal tissues from human aortas (*P<0.05). (**c**–**f**) Four-week-old male ApoE^−/−^ mice were fed with high-fat diet containing 0.15% cholesterol and 20% fat for 16 weeks. They were received a subcutaneous injection of scrambled shRNA (Scr) or RNCR3 shRNA viral vector (R), or PBS. shRNA injection was started at 4 weeks after feeding with high-fat diet. Viral vector was injected once every 2 weeks. Representative en face Oil red O staining in the aortas of PBS-, scrambled shRNA-, and RNCR3 shRNA-injected mice. Scale bar, 0.5 cm. Atherosclerotic lesions quantification in en face aortas was expressed as the percentage of lesions relative to total aortic area (C and D, *n*=6 per group). Representative oil red O staining of aortic sinus in PBS-, scrambled shRNA-, and RNCR3 shRNA-injected mice. Scale bar, 300 *μ*m. Aortic sinus lesion quantification was shown as the change compared with PBS-injected group. *P<0.05 *versus* PBS-injected group (**e** and **f**, *n*=6 per group)

We then determined whether RNCR3 expression is altered during atherosclerosis. The atherosclerotic segments of aortas from apoE^−/−^ mice expressed higher levels of RNCR3 compared with non-atherosclerotic aorta segments of the same ApoE^−/−^ mice, as well as aortas from WT mice (Figure 2a). Compared with the surrounding normal aortic tissue, human aortic atherosclerotic lesion had ~5-fold increase in RNCR3 expression levels (Figure 2b and Supplementary Table S1).

We then determine the role of RNCR3 in the setting of atherogenesis. We found that viral shRNA injection did not induce a detectable immune response, as serum levels of IL-6 and monocyte chemoattractant protein 1 (MCP-1) in mice treated with scrambled shRNA or RNCR3 shRNA did not differ from that of mice injected with PBS alone (Supplementary Figure S1A). The efficacy of RNCR3 inhibition was assessed in several tissues, including lung, heart, liver, kidney, and thoracic aorta. RNCR3 expression was significantly reduced by RNCR3 shRNA injection, but not by scrambled shRNA or PBS (Supplementary Figure S1B). RNCR3 shRNA specifically reduced the levels of RNCR3, but not other lncRNAs, such as MALAT1, TUG1, GAS5, and MIAT (Supplementary Figure S1C).

Quantification of atherosclerotic lesions in thoracic aorta by en face analysis after 4 months of high-fat diet showed that RNCR3 shRNA injection significantly increased atherosclerosis compared with scrambled shRNA-injected or PBS-injected mice (Figures 2c and d). Aortic sinus lesions also significantly increased in RNCR3 shRNA-injected ApoE^−/−^ mice compared with scrambled shRNA-injected or PBS-injected mice (Figures 2e and f).

### RNCR3 knockdown aggravates hypercholesterolemia and inflammatory factor releases

**Figure 3:**
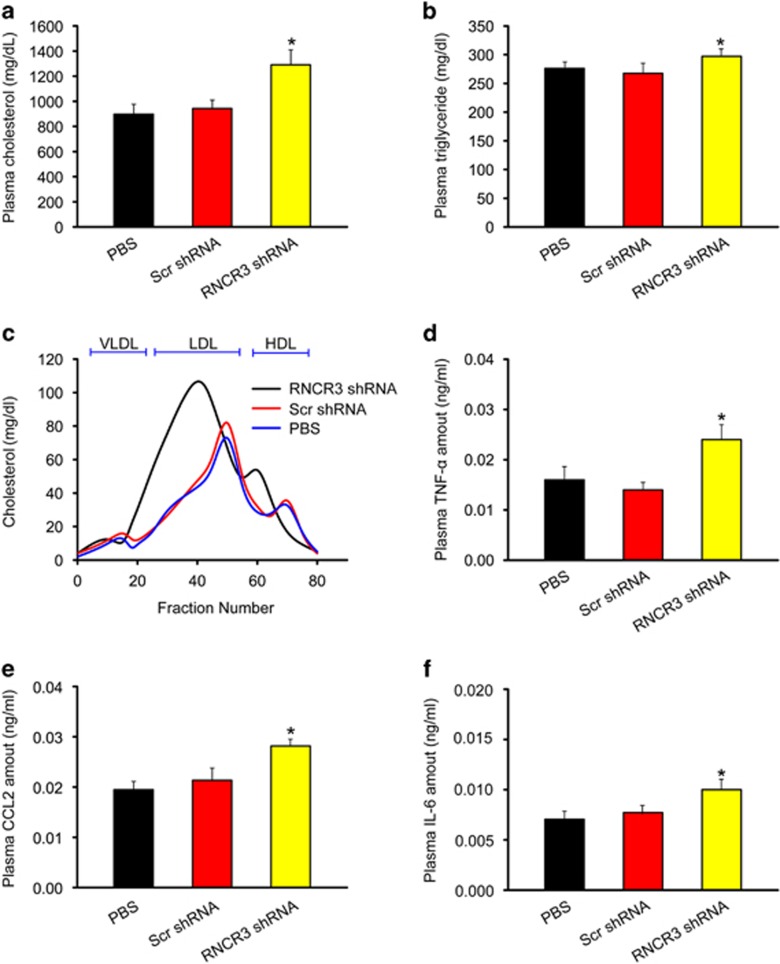
RNCR3 knockdown aggravates hypercholesterolemia and increases inflammatory factor releases. Four-week-old male ApoE^−/−^ mice were fed high-fat diet containing 0.15 cholesterol and 20% fat for 16 weeks. They were received a subcutaneous injection of scrambled shRNA (Scr) or RNCR3 shRNA viral vector (R), or PBS. shRNA injection was started at 4 weeks after feeding high-fat diet. Viral vector was injected once every 2 weeks. (**a**, **b**) Plasma cholesterol and triglycerides levels were detected in PBS-, scrambled shRNA-, and RNCR3 shRNA-injected mice (*n*=6 per group). (**c**) Fast protein liquid chromatographic (FPLC) lipoprotein profiles from the pooled plasma (*n*=6 per group) of PBS-, scrambled shRNA-, and RNCR3 shRNA-injected mice. (**d**–**f**) Plasma levels of TNF-*α*, CCL2, and IL-6 protein from PBS-, scrambled shRNA-, and RNCR3 shRNA-injected mice were quantified by ELISAs (*n*=6 per group). *P<0.05 *versus* PBS-injected group. HDL, high-density lipoprotein; LDL, low-density lipoprotein; VLDL, very low-density lipoprotein

Plasma lipids were analyzed in the ApoE^−/−^ mice after the following treatments (a) PBS-injection; (b) scrambled shRNA-injection; (c) RNCR3 shRNA-injection. Of note, RNCR3 knockdown significantly increased the levels of total cholesterol and triglycerides (Figures 3a and b). Gel-filtration analysis revealed a pronounced elevation of cholesterol in the LDL fraction in RNCR3 shRNA-injected ApoE^−/−^ mice (Figure 3c). These results suggest that RNCR3 knockdown could aggravate hypercholesterolemia *in vivo*.

Atherosclerosis is usually recognized as a hyperlipidemia-induced chronic inflammatory process of arterial wall.^2^ ELISAs revealed that compared with PBS-injected or scrambled shRNA-injected mice, RNCR3 shRNA-injected mice had higher levels of inflammatory factors in blood plasma, such as TNF-α, CCL2, and IL-6 (Figures 3d–f), suggesting that RNCR3 knockdown could aggravate hyperlipidemia-induced inflammation.

### RNCR3 knockdown affects EC and VSMC function *in vivo*

**Figure 4:**
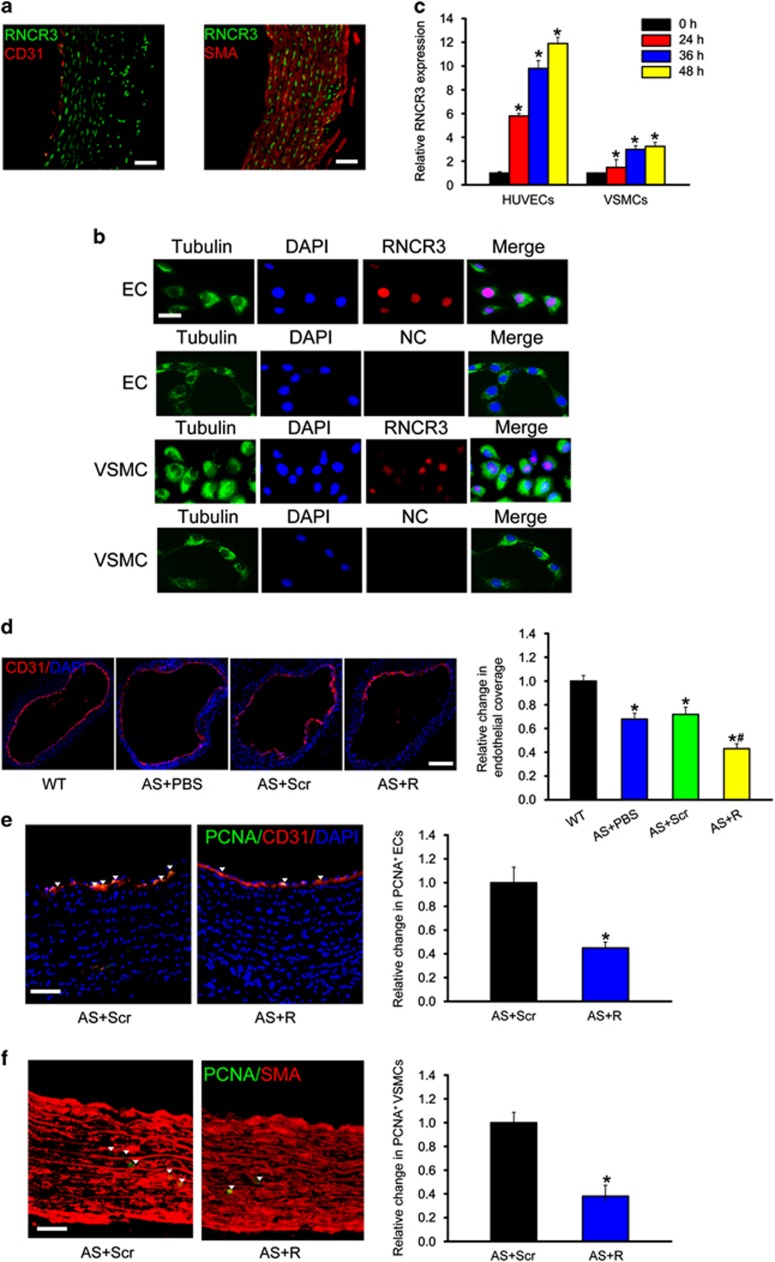
RNCR3 knockdown affects EC and VSMC function *in vivo.* (**a**) *In situ* hybridization for RNCR3 (green) and immunostaining for CD31 (red) or SMA (red) was performed in thoracic aorta of wild-type C57B/6J mice. Scale bar, 50 *μ*m. (**b**) RNA-FISH was performed to detect RNCR3 expression in ECs and VSMCs. Nuclei, blue; RNCR3, red; and Tubulin, green. Tubulin was detected as a cytoplasmic marker to show cell boundary. Scale bar, 20 *μ*m. (**c**) HUVECs or VSMCs were exposed to proatherogenic ox-LDL (25 *μ*g/ml) for the indicated time periods. qRT-PCRs were conducted to detect RNCR3 levels. The data were shown as fold increase compared with untreated group (0 h). *Significant difference compared with untreated group. (**d**–**f**) ApoE^−/−^ mice were fed high-fat diet for 4 weeks, and then injected subcutaneously with RNCR3 shRNA adenovirus for additional 12 weeks (with high-fat diet). The scrambled shRNA or PBS was injected as the controls. Endothelial coverage of carotid artery was determined by CD31 immunostaining (CD31, red; DAPI, blue). *P<0.05 *versus* wild-type (WT) group. ^#^*P*<0.05 AS+Scr shRNA (Scr) *versus* AS+RNCR3 shRNA (R). Scale bar, 200 *μ*m (**d**). Endothelial proliferation of carotid artery was determined by double immunostaining for PCNA and CD31. Scale bar, 50 *μ*m. *P<0.05 *versus* scrambled shRNA-injected group (**e**). Vascular smooth muscle cell proliferation of carotid artery was determined by double immunostaining for PCNA and SMA. Scale bar, 50 *μ*m. *P<0.05 *versus* scrambled shRNA-injected group (**f**)

Based on the above-mentioned results, we knew that RNCR3 is involved in atherosclerosis. We subsequently investigated the potential mechanism. RNA-fluorescent *in situ* hybridization revealed that RNCR3 was mainly expressed in the endothelial cells (co-localization with CD31) and artery smooth muscle cells (co-localization with SMA) (Figure 4a). RNCR3 was constitutively expressed in human umbilical vein endothelial cells (HUVECs) and VSMCs. Notably, RNCR3 transcript was mainly localized in the nuclei (Figure 4b).

We further investigated whether RNCR3 expression is altered upon hypercholesterolemia stress *in vitro*. ox-LDL treatment significantly upregulate RNCR3 expression levels in HUVECs and VSMCs. Notably, greater change for RNCR3 expression was detected in HUVECs than that in VSMCs upon ox-LDL treatment (Figure 4c), implying that RNCR3 in HUVECs has a more prominent role during atherosclerosis.

Hyperlipidemia induces EC injury and apoptosis during atherosclerosis. Damaged ECs can be replaced rapidly by the proliferation of resident ECs, thus preventing frank denudation of the luminal surface.^3^ CD31 staining showed that atherosclerosis resulted in an obvious loss of endothelial coverage. RNCR3 knockdown further decreased endothelial coverage in thoracic aorta (Figure 4d). PCNA/CD31 double staining showed that RNCR3 knockdown reduced endothelial cell proliferation in thoracic aorta (Figure 4e). We also showed that RNCR3 knockdown reduced VSMC proliferation in thoracic aorta (Figure 4f). Collectively, these results indicate that RNCR3 regulates EC and VSMC function *in vivo*.

### RNCR3 knockdown affects EC and VSMC function *in vitro*

We further determined the functional significance of RNCR3 alteration in ECs *in vitro*. We first designed three different RNCR3 siRNAs, and found that RNCR3 siRNA transfection reduced RNCR3 levels in ECs (Supplementary Figure S2A). We selected one RNCR3 siRNA with the greatest silencing efficiency for subsequent function analysis. Moreover, the selected RNCR3 siRNA specially reduced RNCR3 expression but not other lncRNA expression (Supplementary Figure S2B).

**Figure 5:**
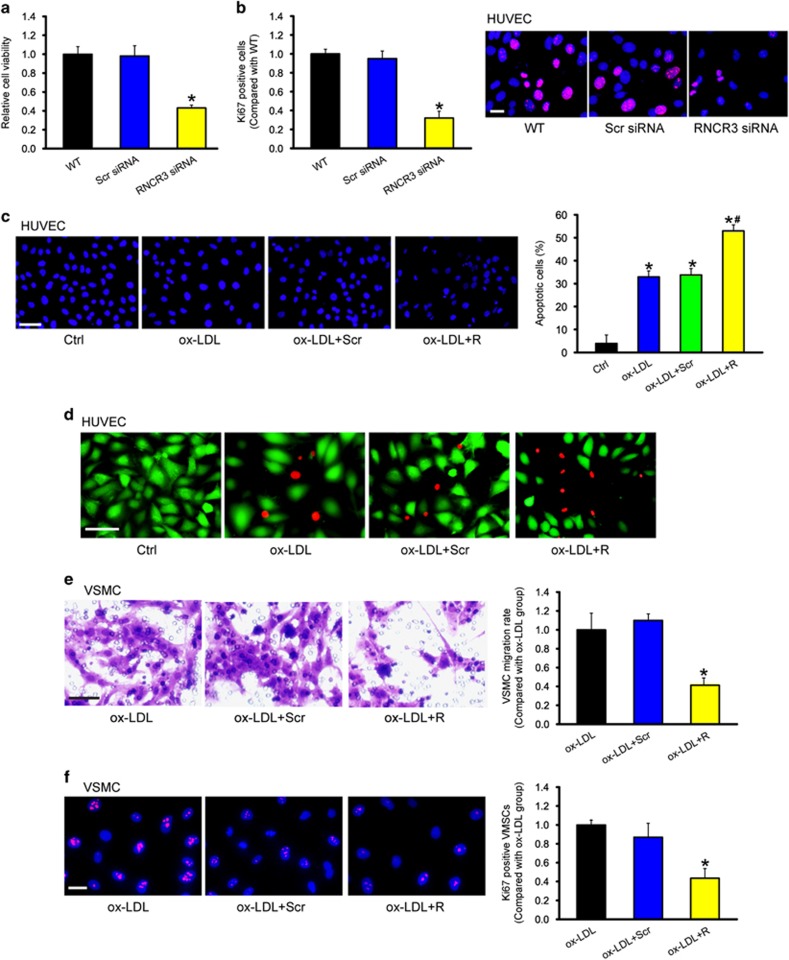
RNCR3 knockdown affects EC and VSMC function *in vitro*. HUVECs were transfected with scrambled (Scr) siRNA, RNCR3 siRNA, or left untreated (WT) for 48 h. Cell viability was detected using MTT method. *P<0.05 *versus* WT group (**a**, *n*=4). Ki67 immunofluorescence staining and quantitative analysis showed that RNCR3 knockdown reduced HUVEC proliferation. Scale bar, 20 *μ*m. *P<0.05 *versus* WT group (**b**, *n*=4). (**c**) HUVECs were transfected with scrambled (Scr) siRNA, RNCR3 siRNA, or left untreated (WT), and then exposed to ox-LDL (25 *μ*g/ml) for 48 h. The group without ox-LDL treatment was taken as the control group (Ctrl). Apoptotic cells were analyzed using Hoechst staining and quantitated. Scale bar, 50 *μ*m. *P<0.05 *versus* Ctrl group; ^#^*P*<0.05 *versus* Ctrl group; ^#^*P*<0.05 AS+Scr shRNA (Scr) *versus* AS+RNCR3 shRNA (R). Dead or dying cells were analyzed using calcein-AM/PI staining. Green, viable cells; red, dead or dying cell. Scale bar, 50 *μ*m (**d**, *n*=4). (**e**, **f**) HUVECs were transfected with scrambled siRNA, RNCR3 siRNA, or left untreated, and then exposed to ox-LDL (25 *μ*g/ml) for 48 h. The medium was collected from these experimental groups, and then co-cultured with VSMCs for 24 h. VSMC migration or proliferation was detected using transwell migration assay or Ki67 staining. A representative image of cell migration (**e**, scale bar, 50 *μ*m, *n*=4) and cell proliferation (**f**, scale bar, 20 *μ*m, *n*=4) and quantification results were shown. *P<0.05 *versus* ox-LDL group

MTT assay showed that RNCR3 knockdown significantly reduced HUVEC viability (Figure 5a). Ki67 immunofluorescence staining showed that RNCR3 knockdown decreased the proliferation of HUVECs (Figure 5b). In response to ox-LDL stress, RNCR3 knockdown accelerated the development of HUVEC apoptosis as shown by increased apoptotic nuclei (condensed or fragmented; Figure 5c) and increased PI-positive cells (dying or dead cells) (Figure 5d).

Communication between ECs and VSMCs has been implicated in the development of atherosclerosis.^20^ We thus investigated whether altered endothelial RNCR3 expression affected the proliferation and migration of VSMCs. HUVECs were transfected with scrambled siRNA, RNCR3 siRNA, or left untreated, and then exposed to ox-LDL for 48 h. After these treatments, the medium was collected from these experimental groups, and then co-cultured with VSMCs. Incubation of VSMCs with the medium collected from RNCR3 knockdown ECs significantly decreased the proliferation and migration of VSMCs (Figures 5e and f), suggesting a critical role of RNCR3 in EC-VSMC communication.

### EC–VSMC communication is mediated by RNCR3-contained exosomes

**Figure 6:**
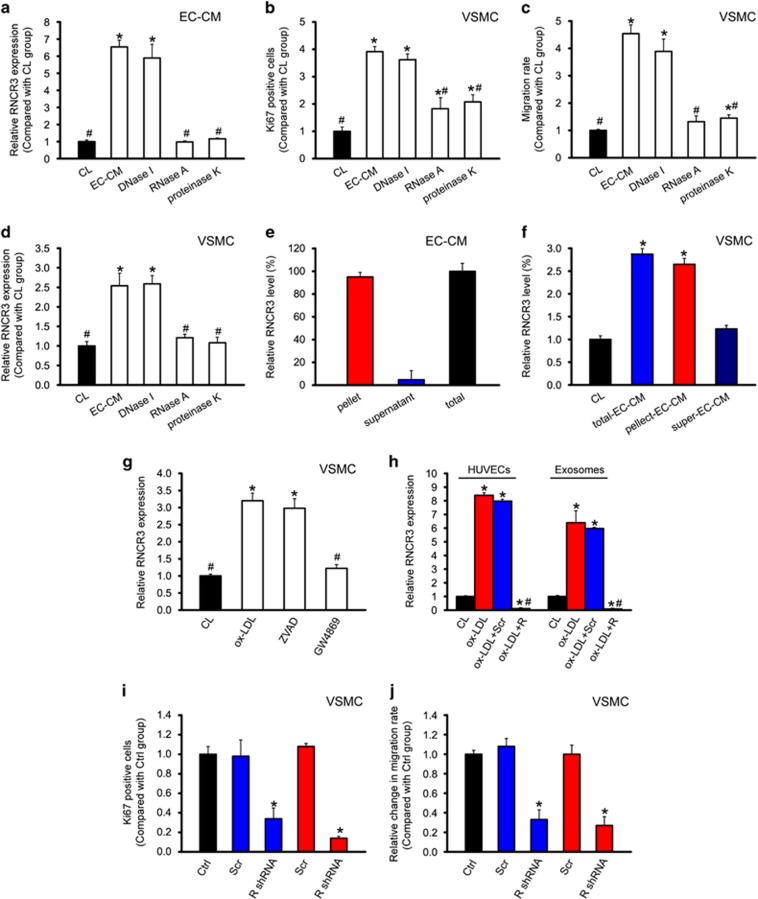
EC–VSMC communication is mediated by RNCR3-contained exosomes. (**a**–**c**) HUVECs were exposed to ox-LDL for 24 h or left untreated (CL). The medium was centrifuged at 1000 × *g* for 10 min to remove cell debris. The supernatant was transferred to a new tube and spun at 120,000 × *g* for 120 min. The pellets were resuspended (designated as EC-CM). EC-CM was incubated with DNase I (1 unit/ml, Invitrogen, Carlsbad, CA, USA) or RNase A (10 *μ*g/ml, Invitrogen) at 37 °C for 15 min, or proteinase K (PK, 20 *μ*g/ml, Invitrogen) at 55 °C for 15 min and then at 95 °C for 5 min to inactivate PK. RNCR3 levels were detected in the control (CL) media or EC-conditioned media (CM) treated with DNase I, RNase A, or PK (**a**). VSMC proliferation (**b**) or migration (**c**) was detected using Ki67 staining or transwell migration assay. *P<0.05 *versus* CL media; ^#^*P*<0.05 *versus* EC-CM group. (**d**) RNCR3 levels in VSMCs incubated with control (CL) media or the EC-conditioned media (CM) treated with DNase I, RNase A, or PK were detected. *P<0.05 *versus* CL media; ^#^*P*<0.05 *versus* EC-CM group. (**e**, **f**) EC-CM was ultracentrifuged to fractionate the components in spin-down pellets (Pellet), or the remaining supernatant (Super). RNCR3 levels were detected in the pellet and supernatant fraction (**e**). RNCR3 levels in VSMCs incubated with control (CL) media, total or fractionated EC-CM for 6 h were detected. *P<0.05 *versus* CL media (**f**). (**g**) HUVECs were treated with apoptosis inhibitor Z-VAD-FMK (ZVAD) or the N-SMase inhibitor GW4869 after ox-LDL treatment for RNCR3 induction. VSMCs were incubated with the vesicles isolated from the above-mentioned HUVECs groups for 12 h. RNCR3 levels in VSMCs were detected. *P<0.05 *versus* CL media; ^#^*P*<0.05 *versus* ox-LDL group. (**h**) HUVECs were transfected with RNCR3 siRNA, scrambled siRNA, or left untreated for 24 h, and then treated with or without ox-LDL (25 *μ*g/ml) for 12 h. RNCR3 levels in HUVECs or exosomes were detected. *P<0.05 *versus* CL media; ^#^*P*<0.05 *versus* ox-LDL group. (**i**,** j**) VSMCs were incubated with the media (CM) derived from ECs transfected with scrambled siRNA or RNCR3 siRNA, or co-cultured with ECs transfected with scrambled siRNA or RNCR3 siRNA for 24 h. VSMC without any treatment was taken as the control group. VSMC proliferation (**i**) or migration (**j**) was detected using Ki67 staining or transwell migration assay. *P<0.05 *versus* Ctrl group

To determine the possible EC-secreted constituents responsible for EC–VSMC communication, we exposed the medium from ECs (EC-CM) after deoxyribonuclease, ribonuclease A (RNase A), or proteinase K treatment. RNase A and proteinase K treatment reduced RNCR3 levels in EC-CM (Figure 6a). The ability of EC-CM to increase VSMC proliferation and migration was abolished by RNase A and reduced by proteinase K but not affected by deoxyribonuclease treatment (Figures 6b and c). These results suggest that RNA and RNA–protein complex was transmitted into VSMCs but not DNA component. We then determine whether EC-secreted constituents affect RNCR3 levels in VSMCs. The ability of EC-CM to increase RNCR3 amount in VSMCs was abolished by RNase A and reduced by proteinase K but not affected by deoxyribonuclease (Figure 6d).

We separated extracellular vesicles from HUVEC-derived CM by ultracentrifugation and detected RNCR3 levels in vesicle-containing pellets and vesicle-poor supernatant. Approximately 95% of extracellular RNCR3 was present in the pellet, and 5% was present in the supernatant (Figure 6e). Treatment of VSMCs with the pellet led to increased RNCR3 levels in VSMCs comparable to that resulting from the unseparated EC-CM, but increased RNCR3 levels were not observed in VSMCs treated with the supernatant (Figure 6f).

Extracellular vesicles may derive from the remnants of apoptotic cells (apoptotic bodies) or actively exported exosomes. Pharmacological inhibition of sphingomyelinase, which was shown to inhibit exosome generation, attenuated the transfer of RNCR3 to VSMCs, whereas inhibition of apoptosis did not affect RNCR3 transfer (Figure 6g), indicating that the transfer is mediated by actively formed vesicles, probably exosomes rather than apoptotic bodies.

Exosomes carries functional molecular and mediates cell–cell communication.^20^ We isolated exosomes from the medium of RNCR3 siRNA and scrambled siRNA-transfected HUVECs. ox-LDL treatment significantly upregulated RNCR3 levels in HUVECs and exosomes, while RNCR3 levels were significantly reduced after RNCR3 siRNA transfection in HUVECs and exosomes (Figure 6h).

We further investigated whether exosome-derived RNCR3 has similar functional effect on VSMCs as shown in co-culture experiments. HUVECs-derived exosomes induced VSMC proliferation and migration in a similar manner to that observed in co-culture experiments (Figures 6i and j).

### RNCR3 regulates endothelial cell function by acting as a ceRNA

**Figure 7:**
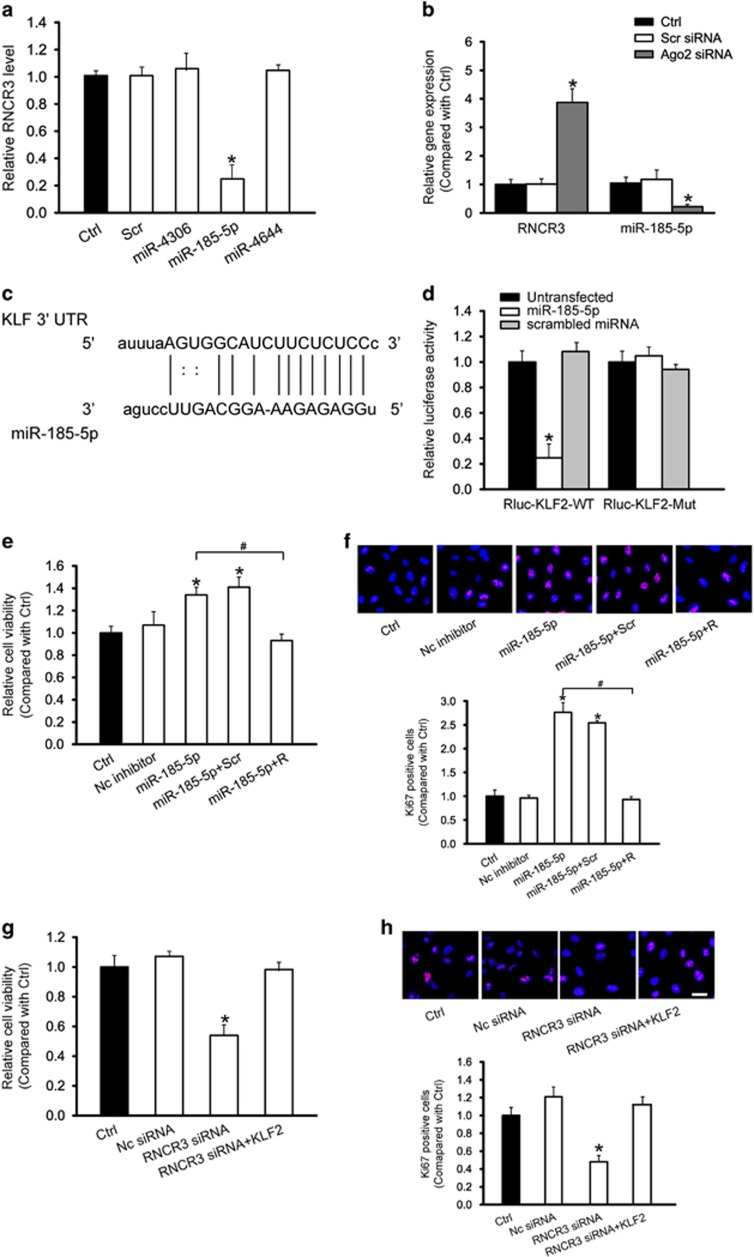
RNCR3 regulates endothelial cell function by acting as a ceRNA. (**a**) HUVECs were transfected with different miRNA mimics, or left untreated (Ctrl) for 48 h. qRT-PCRs were conducted to detect RNCR3 levels. The data was expressed as relative change compared with Ctrl group. *P<0.05 *versus* Ctrl group. (**b**) HUVECs were transfected with Ago2 siRNA, scrambled siRNA, or left untreated (Ctrl). miR-185-5p or RNCR3 levels were detected using qRT-PCRs. *P<0.05 *versus* Ctrl group. (**c**) KLF2 was predicted as a target gene of miR-185-5p using TargetScan. The position of miR-185-5p binding site on KLF2 was shown. (**d**) KLF2 (RLuc-KLF2-WT) or mutant (RLuc-KLF2-Mut) was co-transfected with miR-185-5p mimic, scrambled miRNA mimic, or left untreated. Luciferase activity was detected using the dual luciferase assay. *P<0.05 *versus* untransfected group. (**e–h**) HUVECs were treated as shown. Cell viability was detected using MTT assay (**e, g**). A representative image for Ki67 staining along with quantification analysis data was shown. Scale bar, 20 *μ*m (**f**, **h**). *P<0.05 *versus* Ctrl group. ^#^Significant difference between the marked groups (^#^*P*<0.05)

RNCR3 is a lncRNA transcribed from the intergenic regions of the genome. Numerous studies have shown that lincRNAs act as competing endogenous RNAs (ceRNAs) by decreasing targeting concentration of microRNA (miRNA), ultimately resulting in the derepression of other messenger RNAs (mRNAs) having the common miRNA response elements.^17, 21^ We thus employed the StarBase v2.0 to search for the potential miRNA recognition elements on RNCR3.^22^ miR-4306, miR-185-5p, and miR-4644 was predicated as potential miRNA targets of RNCR3. RNCR3 levels were significantly reduced by miR-185-5p mimic, but not by other miRNA mimics (Figure 7a). Ago2 is a key component of RNA-induced silencing complex that binds miRNA complexes to mRNA targets.^23^ We studied whether RNCR3 expression is regulated by miRNAs via Ago2 knockdown *in vitro*. Ago2 knockdown resulted in a significant increase in RNCR3 expression, whereas miR-185-5p stability was impaired by Ago2 knockdown (Figure 7b).

We employed TargetScan database to predict the potential mRNA targets of miR-185-5p. Among these putative targets, we focused on KLF2, a transcriptional factor conferring an endothelial vasoprotective phenotype^24^ (Figure 7c). The 3′-UTR of KLF2 was fused into luciferase coding region (RLuc-KLF2-WT) and transfected into HUVECs with miR-185-5p mimic or negative control mimic. Luciferase assays revealed that KLF2 was a target of miR-185-5p. The use of mutant derivatives (-Mut) in the miRNA recognition site further verified the specificity of inhibitory effect (Figure 7d).

We then determined whether RNCR3-miR-185-5p is involved in regulating EC function. miR-185-5p inhibitor transfection increased the viability and proliferation of HUVECs (Figures 7e and f), whereas RNCR3 knockdown partially abolished this effect. We also investigated whether the RNCR3-KLF2 cross-talk is involved in regulating EC function. RNCR3 knockdown decreased the viability and proliferation of HUVECs, whereas KLF2 overexpression partially reversed the effect of RNCR3 knockdown on HUVEC function (Figures 7g and h).

As RNCR3, miR-185-5p, and KLF2 constitutes a regulatory network, we then investigated whether KLF2 co-expressed with RNCR3 in mouse and human atherosclerotic lesions. The atherosclerotic segments of aortas from apoE^−/−^ mice expressed higher levels of KLF2 compared with non-atherosclerotic aorta segments of the same ApoE^−/−^ mice, as well as aortas from WT mice (Supplementary Figure S3A). Compared with surrounding normal aortic tissue, human aortic atherosclerotic lesion had higher KLF2 expression levels (Supplementary Figure S3B), suggesting that KLF2 has similar expression pattern as RNCR3. We also showed that RNCR3 knockdown resulted in a significant reduction in KLF2 levels (Supplementary Figure S3C). Overexpression of miR-185-5p significantly reduced the expression of KLF2 and RNCR3 (Supplementary Figure S3D). Collectively, these results further suggest there is a cross-talk between KLF2 and RNCR3 through interacts with miR-185-5p.

## Discussion

More than 90% of human genome is transcribed, whereas protein-coding genes only represents <3% of the total genomic sequence. Tens of thousands of lncRNAs is transcribed.^25, 26^ To date, the vast majority of lncRNAs have yet to be characterized thoroughly. Here we show that RNCR3 deficiency accelerates atherosclerosis development in ApoE^−/−^ mice. RNCR3 could regulate EC and VSMC function *in vitro* and *in vivo*. Mechanistically, RNCR3 acts as a ceRNA, and forms a feedback loop with KLF2 and miR-185-5p to elicit atheroprotective properties to the endothelium.^27^ RNCR3 also serves as a paracrine mediator secreted by the EC to act on the co-cultured SMC to modulate its function toward atherogenic phenotype. Thus, RNCR3 intervention may provide a promising strategy to combat atherosclerosis.

Blood vessels deliver oxygen and nutrients to every part of the body. The vascular endothelium is quiescent under physiological conditions. Blood vessels possess the capacity to rapidly form new vasculature in response to injury or in pathological conditions, such as hypoxia, oxidative stress, and inflammatory stress.^28^ The key players in angiogenesis are the blood vessel lining ECs.^29^ Atherosclerosis or exogenous VEGF stimulation causes hypoxia, oxidative stress, or inflammatory stress in the retina and cornea, thereby leading to pathological angiogenesis. RNCR3 knockdown significantly reduces the development of angiogenesis, suggesting a key role of RNCR3 in EC function maintenance. Atherosclerosis is initiated by EC dysfunction of vessel wall. Damaged ECs are usually replaced by the proliferation of resident ECs.^2^ RNCR3 knockdown could reduce EC proliferation and impair EC regeneration in injured arteries, suggesting that RNCR3 knockdown reduces EC proliferation and contributes to EC apoptosis upon stress. Ocular angiogenesis is an important cause for severe loss of vision. The new blood vessels could increase vascular permeability and vascular fragility, leading to retinal edema, hemorrhage, fibrovascular proliferation with tractional and rhegmatogenous retinal detachment.^30^ By contrast, the replicative regeneration of arterial ECs could prevent atherosclerotic lesion formation and limit atherosclerosis. Human tissues exhibit distinct characteristics despite differentiating from a common origin to fulfill the different needs. Thus, a study on tissue-specific transcriptional regulation is required. Drug development and safe use should consider the distinct efficacy in different tissues.^31^

Based on *in vivo* and *in vitro* evidence, we speculate that RNCR3 upregulation is a potential stress response during atherosclerosis. Atherosclerosis develops preferentially at sites where disturbed laminar flow compromises EC function.^3, 32^ Hyperlipidemia induces EC injury and apoptosis, leading to a dysfunctional arterial endothelium. Endothelial integrity is usually maintained through the replacement of damaged ECs with proliferating and healthy ECs. Loss of functional integrity of the endothelium has an integral role at all stages of atherosclerosis from lesion initiation to plaque rupture.^33, 34^ We show that atherosclerosis or ox-LDL treatment leads to a marked increase in RNCR3 level. RNCR3 knockdown impairs EC regeneration in injured arteries, decreases EC proliferation, and indirectly affects VSMC proliferation and migration. Thus, RNCR3 knockdown accelerates atherosclerosis development, and ultimately aggravates hypercholesterolemia and inflammatory response.

We demonstrate that endothelial expression and secretion of lncRNA-RNCR3 regulates VSMC proliferation and migration *in vitro*. Increasing studies have revealed the cross-talk between ECs and VSMCs by extracellular vesicles.^20, 35, 36^ We show that EC–VSMC communication is mediated by extracellular vesicles, which are enriched in RNCR3. Extracellular vesicles may derive from the remnants of apoptotic cells (apoptotic bodies) or actively exported exosomes. We show that RNCR3 transfer is mediated by exosomes rather than apoptotic bodies. ECs-derived exosomes induces VSMC proliferation and migration. Stabilization of vessel architecture is not only guided by ECs, but also guided by signaling from other cell types, such as VSMCs. In normal vasculature, VSMCs are important for the stabilization of ECs and mediate EC survival together with the maturation of the vessels. In contrast, VSMCs are usually absent in or have loose associations with ECs under pathological condition, leaving most of the new blood vessels immature.^36, 37^ The pathological signals may also be transferred from one cell type to another. On the basis of our data, it can be speculated that RNCR3 is upregulated to maintain the pro-angiogenic phenotype of ECs, while RNCR3 level in the surrounding VSMCs has to be low due to inefficient RNCR3 transfer from ECs to VSMCs or less RNCR3 induction in VSMCs. The imbalance between EC and VSMC function maintenance could ultimately lead to vascular injury. Therefore, vesicle-mediated transfer of RNCR3 may provide a promising strategy to combat atherosclerosis.

Long intergenic non-coding RNAs (lincRNAs) are located between protein-coding genes. Some lincRNAs are cis- and trans-regulators of gene activity by functioning as scaffolds for chromatin-modifying complexes and nuclear bodies or as enhancers and mediators of long-range chromatin interactions.^38, 39^ Some lincRNAs function as ceRNA in modulating the concentration and biological functions of miRNAs,^21^ such as RoR^40^ and linc-MD1.^41^ These ceRNAs generally share miRNA-response elements with the transcripts of several important genes and prevent these mRNAs from being degraded. RNCR3 is also a lincRNA. RNCR3 may have a regulatory role in atherosclerosis through ceRNA cross-talk and competition. We show that lncRNA-RNCR3 communicates with and co-regulates KLF2 by competing for binding to miR-185-5p. During atherosclerosis, RNCR3 is significantly upregulated, which alleviates miR-185-5p repression effect, thereby upregulating the level of miR-185-5p target gene, KLF2. This regulatory loop maintains a relative balance in endothelial function to resist proatherogenic stress. miR-185-5p is shown as a post-transcriptional regulator. LncRNA-RNCR3 functions as a genome regulator at the transcriptional level. The regulatory loop also integrates transcriptional and post-transcriptional regulatory network involved in atherosclerosis.

KLF2 is a critical regulator of endothelial and monocyte/macrophage proinflammatory action. KLF2 expression is induced by laminar shear stress and inhibited by proinflammatory cytokines in endothelial cells.^24, 42^ Sustained overexpression of KLF2 induces endothelial nitric oxide synthase and thrombomodulin expression, and reduces cytokine-mediated activation of proinflammatory genes.^43^ Hemizygous deficiency of KLF2 increases diet-induced atherosclerosis in apolipoprotein E-deficient mice. KLF2 has been viewed as a candidate atheroprotective factor.^44^ We show that lncRNA-RNCR3 functions as a ceRNA to regulate KLF2 levels by sponging miR-185-5p in endothelial cells. RNCR3 overexpression may become a sink for miR-185-5p, thereby affecting the derepression of KLF2. KLF2 release could have an atheroprotective role during atherogenesis. The ceRNA regulatory network, RNCR3/miR-185-5p/KLF2, would provide a novel insight into gene regulatory network in atherosclerosis.

In conclusion, we report that RNCR3 have an atheroprotective role in atherogenesis. RNCR3 knockdown contributes to EC and VSMC dysfunction, and aggravates atherosclerosis. Hence, upregulation of RNCR3 level seems as a novel therapeutic strategy to protect against hypercholesterolemia-induced EC and VSMC dysfunction and atherosclerosis development.

## Materials and Methods

### Ethics statement

Animal experiments were approved by the Animal Care and Use Committee of Nanjing Medical University, and were handled in accordance with the ARVO Statement for the Use of Animals in Ophthalmic and Vision Research. All animals were maintained under standard 12 h light–dark cycles. The surgical specimens were collected according to the Declaration of Helsinki. All patients were obtained the informed consent before inclusion.

### Cell culture

HUVECs (Promocell, Heidelberg, Germany) were cultured in endothelial basal medium supplemented with hydrocortisone, bovine brain extract, epidermal growth factor, gentamycin and 10% fetal bovine serum at 37 °C in a humidified atmosphere of 95% air and 5% CO_2_. Human VSMCs (ATCC, Rockville, MD, USA) were cultured in Kaighn's modified Hams F12 medium supplemented with 10 FBS and 50% VSMC growth medium.

### Plasma lipid measurement

Mice were fasted for 6 h before blood sample collection. Plasma was separated by centrifugation and stored at −80 °C. Total plasma cholesterol and triglyceride concentrations were enzymatically measured using kits (Thermo Trace Ltd., Melbourne, Australia). Lipoprotein profiles were analyzed using the microFPLC column (30 × 0.32 cm Superose 6B; GE Healthcare) coupled to a system for online separation and subsequent detection of cholesterol.^45^

### Lipid staining of aortic valve and aorta

Lipid was stained using Oil Red O (Sigma, St. Louis, MO, USA). Aortic valve staining was carried out using the frozen sections after perfusion, fixation (4% paraformaldehyde), dehydration (30% sucrose) and embedding (O.C.T. Compound). Ten-micrometer thick frozen sections were used to detect the area of atherosclerotic lesion in aortic sinus. En face Oil red O staining was conducted to detect lipid distribution in the aortic arch and thoracic aorta. The vessels were fixed in 4% paraformaldehyde and stained in Oil Red O for 20 min. Plaque area was calculated as the percentage of total specimen area.

### Preparation of exosomal fraction

The medium of HUVECs was collected and centrifuged at 3 000 × *g* for 15 min. The supernatant was filtered through 0.22-*μ*m PVDF filter (Millipore, Bedford, MA, USA). The appropriate volume of Exoquick Exosome Precipitation Solution (System Biosciences, Mountain View, CA, USA) was added to the filtered culture medium and mixed well by inverting. After 10 h refrigeration, the mixture was centrifuged at 2 000 × *g* for 30 min. The supernatant was removed by aspiration. Exosome pellets were re-suspended using 500 *μ*l of the serum-free AIM V medium (Life Technologies, Grand Island, NY, USA).

### Statistical analysis

Statistical analyses were conducted using GraphPad Prism 5 software. Data was tested for the normality by the D'Agostino–Pearson omnibus normality test and similar variance by *F-*test. Comparison between any two groups was by two-tailed unpaired *t*-test for normally distributed data or non-parametric Mann–Whitney test for non-normally distributed data. Multiple group comparison was done by one-way analysis of variance for data with normal distribution. Kruskal–Wallis test was used for data with non-normal distribution. A probability value *P*<0.05 was considered statistically significant.

## Abbreviations

lncRNA: long noncoding RNA
ELISA: enzyme-linked immunosorbent assay
shRNA: short hairpin RNA
siRNA: short interfering RNA
VEGF: vascular endothelial growth factor
EC: endothelial cell
VSMC: vascular smooth muscle cell
RNCR3: retinal non-coding RNA3
KLF2: kruppel-like factor 2
ceRNA: competing endogenous RNA
miRNA: microRNA
