# Supplementary material for: Role of long non-coding RNA-RNCR3 in atherosclerosis-related vascular dysfunction
Source: Cell Death Dis. 2016 Jun 2;7(6):e2248–. doi: 10.1038/cddis.2016.145 (PMC5143375; doi:10.1038/cddis.2016.145)
Supplement: Supplementary Information [file cddis2016145x1.doc]

**Supplementary figure legend**

**Fig. S1: RNCR3 shRNA injection down-regulates RNCR3 expression level in ApoE^-/-^ mice**

(A-C) ApoE^-/-^ mice were fed with high-fat diet for 4 weeks, and then injected subcutaneously with RNCR3 shRNA adenovirus. Scrambled shRNA or PBS was injected as the controls. To maximize virus delivery, mice were injected twice during the first week, then once weekly thereafter, for a total of 16 weeks. ELISAs were conducted to the serum levels of IL-6 and MCP-1 (A). qRT-PCRs were conducted to detect RNCR3 levels in lung, heart, liver, kidney, and thoracic aorta (B). qRT-PCRs were conducted to detect the expression of long non-coding RNAs, including MALAT1, RNCR3, MIAT, TUG, and GAS5 (C). The data was shown as fold increase compared with PBS-injected group. **P*<0.05 versus PBS-injected group.

**Fig. S2: RNCR3 siRNA transfection reduces RNCR3 level but not other lncRNA level**

(A) HUVECs were transfected with scrambled siRNA (Scr), RNCR3 siRNA1, RNCR3 siRNA2, RNCR3 siRNA3, or left untreated (WT) for 48 h. qRT-PCRs were conducted to detect RNCR3 expression. The data was shown as fold increase compared with WT group. **P*<0.05 versus WT group. (B) HUVECs were transfected with RNCR3 siRNA for 48 h. qRT-cPCRs were conducted to compare relative expression change of long non-coding RNAs, including RNCR3, MIAT, MALAT1, GAS5, and TUG1, before and after transfection. **P*<0.05 versus before transfection group.

**Fig. S3: KLF2/RNCR3/ miR-185-5p constitutes a regulatory network**

(A) KLF2 expression in the aorta of 5-month old male ApoE^-/-^ and C57B/6J mice was determined by qRT-PCRs and normalized to expression of GAPDH. The data was expressed as relative mRNA level compared with the average expression in wild-type group (WT). WT: wild type C57B/6J mice. E/L-lesion and E/L-normal: aorta segments with atherosclerotic lesions or without lesion (normal) from apoE^-/-^ mice, respectively (**P*<0.05). (B) Expression levels of KLF2 were detected in the atherosclerotic lesions from human aortas and non-lesional aortic intimal tissues (**P*<0.05). (C) Four-week-old male ApoE^-/-^ mice were received a subcutaneous injection of scrambled shRNA (Scr) or RNCR3 shRNA viral vector (R), or PBS for 2 weeks. KLF2 expression was determined by qRT-PCRs and normalized to expression of GAPDH (**P*<0.05). (D) Four-week-old male ApoE^-/-^ mice were received a subcutaneous injection of scrambled mimic (Scr) or miR-185-5p mimic, or PBS for 2 weeks. KLF2 and RNCR3 expression was determined by qRT-PCRs and normalized to expression of GAPDH (**P*<0.05).

**Supplementary materials and methods**

**RNCR3 gene silencing**

Three individual siRNAs (siRNA RNCR3-1, siRNA RNCR3-2, and siRNA RNCR3-3) and negative control siRNA were purchased from Shanghai GenePharma Co., Ltd (China). Target sequences for RNCR3 siRNAs were as follows: siRNA1, 5’- GCTCATTAATTTCCAGGTG-3’, siRNA2, 5’- CGTCCACATTGACGAGCCT-3’, and siRNA3, 5’- GCTCTATCGGAGTTGAAAG-3’. The negative siRNA was purchased as scrambled sequence with no homology within the mouse or human genome. siRNA duplexes were transfected into cells two times (48 h) within a gap of 24 h at a final concentration of 100 nM using Lipofectamine RNAimax reagent (Life Technologies) according to the manufacturer's instruction.

**RNCR3 shRNA adenoviral construction**

Three different RNCR3 RNAi target sequence is shown below:

5’- GCAACCAGGATCCTTTAAAGG-3’；

5’- GCTCCCTCACTTCACAAATGG-3’；

5’-GCCCTTCTCTGCCCATCAATA-3’.

A scrambled RNAi without any other match in the mouse genomic sequence was used as the control (5’-CCGATCTGACATGACTGCG-3’). The adenoviruses harboring these RNAi constructs were generated using the BLOCK-iT™ Adenoviral RNAi Expression System (Life Technologies) according to the instruction. Packaging and amplification of Adenoviruses was conducted in HEK293T cells.

**Intravitreal injection**

AopE^-/-^ mice were anesthetized with 2% isoflurane, and a drop of 0.5% proparacaine was administered as a topical local anesthesia. A fine glass micropipette connected to a 10 µl Hamilton glass syringe was inserted through the incision in the cornea and slid between the iris and the lens into the posterior chamber of the eye. Each eye received about 3 µl adenovirus. Injections were administered slowly over approximately one minute and monitored with a stereo microscope. Injections were conducted once a week for the required time periods.

**In vivo** **RNCR3 inhibition in ApoE^-/-^ mice**

Four-week-old male ApoE^-/-^ mice were fed high-fat diet containing 0.15% cholesterol and 20% fat for 16 weeks. shRNA injection was started at week 4 after high-fat diet. These mice were injected intraperitoneally at a dose of 50 μl/g ([Virus](javascript:void(0);) [titer](javascript:void(0);): 1.0 × 10^13^ viral genomes/ml) of shRNA adenoviral vector. Scrambled shRNA-injected or PBS-injected mice were used as the control group. The body weight of the mice was measured throughout the experimental period.

**Quantitative reverse transcription-PCR (qRT-PCR)**

Total RNAs were extracted using TRIzol reagent (Life Technologies). The quality and purity of RNA was controlled spectrophotometrically and estimated by electrophoresis. 1 μg total RNA was DNase-I treated (Life Technologies) and reversely transcribed into cDNA using the SuperScript First-Strand Synthesis Kit (Takara). qPCRs were performed using cDNA templates in a PikoReal Real-Time PCR System (Thermo Scientific). For qPCR reactions, 30-50 µg cDNA was added to 20 µl PCR reaction mixture containing 10 µl of 2× Power SYBR Green PCR Master Mix and 0.5 µl of each primer (20 pmol). Comparative C_t_ method was used to detect target gene expression in test samples relative to the control samples. Non-template controls were included to verify the method and specificity of the primers. GAPDH expression was detected as the internal control. The primer sequences were shown below:

Mouse RNCR3

Forward primer: 5’-CCAACCTGCAGTGGATGGAT-3’;

Reverse primer: 5’-TCCTGCCCCTCCTCCATTTA-3’;

Human RNCR3

Forward primer: 5’-CTGGAGTCCCAGAAGGCAAG-3’;

Reverse primer: 5’-AAAGCTACTGGTTTCGGGGG-3’.

qPCRs were also performed using kits for qPCR of U6 and mature miR-185-5p (Genpharma, Shanghai, China), according to the manufacturer's instructions. U6 RNA expression was detected as the internal control.

**Hoechst staining**

After the required treatment, cells were fixed in 4% formaldehyde for 15 min at room temperature, and then permeabilized using Triton-X 100 for 10 min. After three times wash, these cells were stained with Hoechst 33342 (100 μg/ml) for 10 min, and then washed by PBS buffer. Stained nuclei were observed using an Olympus IX-73 microscope.

**Cell viability assay**

Cell viability was detected using 3-(4, 5-dimethylthiazol-2-yl)-2, 5- diphenyl-tetrazolium-bromide assay (MTT). Briefly, cells were plated at a density of 1×10^4^ cells per well in 96-well plates. After the required treatment, they were incubated with MTT (0.5 mg/ml) at 37^o^C for 3 h. DMSO solution (100 mM) was finally added to dissolve formazan crystals. The absorbance was measured at absorbance 570 nm using a microplate reader (Molecular Devices).

**Immunofluorescence**

HUVECs or VSMCs were fixed in 4% formaldehyde for 15 min at room temperature, and then permeabilized using Triton-X 100 for 10 min. Nonspecific binding was blocked by incubation with 2% bovine serum albumin (Sigma-Aldrich) for 30 min. They were probed with the primary antibodies followed by incubation with the fluorescent secondary antibodies (Life Technologies). DAPI was used for nuclear staining. Images were acquired using an Olympus IX-73 microscope. As a negative control, normal mouse IgG fraction was used as the primary antibody.

The carotid arteries were fixed with 4% paraformaldehyde at 4°C for 12 h. They were then transferred to 30% sucrose solution for 12 h, embedded in Tissue-Tek OCT compound (Miles), and cut into 10 μm cryo-sections. Non-specific binding was blocked using serum free protein block for 1 h at room temperature. The cryo-sections were stained with CD31 (1:100, Abcam) or SMA (1:200, Abcam) for 24 h at 4 ^o^C to detect endothelial cells or smooth muscle cells, and then stained with PCNA antibody (1:200, Abcam) for 24 h at 4 ^o^C to mark proliferating cells.

**Enzyme-linked immunosorbent assay (ELISA)**

After the required treatment, the culture medium of HUVECs and blood plasma of mice were obtained and centrifuged at 500 g for 15 min, and stored at -80°C. IL-6, MCP-1, TNF-α, and CCL2 levels were quantified using the commercial ELISA kits (BD Pharmingen).

**Calcein-AM and propidium iodide (PI) double staining**

Calcein-AM and PI double staining was used to detect viable and dead cells. Calcein- AM is cell membrane permeable and stains the viable cells, whereas PI is cell membrane impermeable and stains the dead cells. After the required treatments, HUVECs were fixed with 4% formaldehyde for 15 min, and then stained using Calcein-AM solution (10 µmol/L) for 15 min. After washing with PBS buffer, these cells were stained with PI solution (10 µmol/L) for 10 min. The viable cells were observed using a 490 nm excitation filter. The dead cells were observed using a 545 nm excitation filter.

**Transwell cell migration assay**

To determine the migration of VSMCs, VSMCs were placed in the upper chamber of a Transwell (5×10^4^ cells per chamber, pore size 8 μm, Corning) coated with fibronectin (1 μg/ml). The chamber was placed in a 24-well culture dish containing the media from treated endothelial cells. VSMCs were allowed to migrate for 6 h at 37°C in a humidified atmosphere containing 5% CO_2_. Non-migrated VSMCs were scraped off the upper surface of the membrane with a cotton swab. The migrated cells remaining on the bottom surface were counted after staining with crystal violet, and observed using an Olympus IX-73 microscope.

**RNA fluorescence in situ hybridization**

To detect RNCR3 expression distribution, HUVECs or VSMCs were fixed in 4% paraformaldehyde for 15 min, and then permeabilized with 1% Triton X-100 for 10 min. They were rinsed in 2×SSC prior to hybridization. Hybridization was performed at 37°C for 6 h using cDNA probe. Slides were counterstained with Tubulin antibody to show cell boundary, and stained with DAPI to show the nuclei.

Thoracic aorta of C57BL/6J mice was fixed with 4% paraformaldehyde at 4°C for 12 h. They were then transferred to 30% sucrose solution for 12 h, embedded in Tissue-Tek OCT compound (Miles), and cut into 10 μm cryo-sections. The sections were immersed in pre-hybridization buffer containing 50% formamide, 5×Denhardt’s solution, and 5×SSC (1×SSC: 150 mM NaCl, 15 mM sodium citrate, pH 7.0) for 3 h. The sections were then hybridized with RNCR3 at 62°C for 6 h. Slides were washed, incubated in RNase A (20 mg/ml) at 37°C for 30 min, and then stained with SMA or CD31 antibody to show artery smooth muscle cells and endothelial cells. Slides were finally mounted and observed using an Olympus IX-73 microscope.

**Luciferase reporter assay**

The 3’-untranslated regions (UTR) of KLF2 was cloned into the downstream of the firefly luciferase gene in pGL3 vector (Promega). The 3’-UTR without miR-185-5p binding site (Delete eight nucleotides “UUCUCUCC”) was constructed to generate pGL3-KLF2 mutant. For luciferase assays, cells were pre-plated in 24-well plates, and then transfected with either wild-type or mutant construct with and without miRNA mimic or negative control mimic. Forty-eight hours after transfection, firefly and Renilla luciferase activities were detected using the Dual-Glo Luciferase Assay System (Promega). Renilla luciferase activity was normalized to firefly luciferase activity.

**Retinal trypsin digestion assay**

The eyes were fixed in 4% paraformaldehyde for 24 h, equatorially bisected, and the entire retina was removed. The retinas were incubated with 3% trypsin in 0.1 M Tris, 0.1 M maleic acid containing 0.2 M NaF for 1 h at 37°C. Non-vascular tissues were carefully brushed away. Retinal vasculature was then transferred and flat-mounted onto the clean glass slides for periodic acid-Schiff (PAS) and hemotoxylin staining. The number of acellular capillaries per square millimeter of retina was quantified.

**Detection of retinal vascular leaky using Evans blue**

Under deep anesthesia, the right jugular vein and right iliac artery were cannulated, and filled with heparinized saline. Evans blue was injected through the jugular vein (45 mg/kg). Subsequently, at 15 min intervals, 0.1 ml blood was drawn from the iliac artery for 2 h to obtain time-averaged plasma Evans blue concentration. After the dye circulated for 2 h, the chest cavity was opened and perfused. The retinas were carefully dissected away and dried for 6 h. Evans blue was extracted by incubating each retina in 120 μl formamide (Sigma) for 18 h at 70 °C. The supernatant was filtered at 3,000 rpm for 2.5 h and 50 μl of the filtrate was used for triplicate spectrophotometric measurement. Background-subtracted absorbance was detected by measuring each sample at both 620 nm, the absorbance maximum for Evans blue in formamide, and 740 nm, the absorbance minimum. The concentration of Evans blue was calculated from a standard curve and normalized to the dry weight of retina.

**Alkali-induced corneal neovascularization**

A 2.5-mm-diameter circular filter disc was incubated with 1 mol/L NaOH for 60 seconds. The filter disc was placed on the corneal surface for 30 seconds in anesthetized 4-month old male C57BL/6J mice. The ocular surface was then irrigated with 10 ml physiological saline. The subsequent vascular response was measured in a masked manner by slit-lamp biomicroscopy.

**Clinical inclusion criteria**

The coronary artery disease (CAD) group contained patients with greater than 80% coronary artery stenosis by coronary angiography. The control group contained patients without clinically significant coronary artery occlusion by coronary angiogram. Patients with diagnoses of hypertension or Type I/II diabetes were excluded. All procedures were approved by Nanjing Medical University Ethics Committee, and written informed consent was received from all participants.
