# Supplementary material for: Role of long non-coding RNA-RNCR3 in atherosclerosis-related vascular dysfunction
Source: Cell Death Dis. 2016 Jun 2;7(6):e2248–. doi: 10.1038/cddis.2016.145 (PMC5143375; doi:10.1038/cddis.2016.145)
Supplement: Supplementary Table 1 [file cddis2016145x2.doc]

**Table S1: Control and coronary artery patient characteristics**

|  | **Number** | **Gender** | **Age** | **Coronary**  **angiography** | **Hypertension** | **Diabetes** |
| --- | --- | --- | --- | --- | --- | --- |
| **Patients** |  |  |  |  |  |  |
|  | 1 | male | 65 | >80 | negative | negative |
|  | 2 | female | 57 | >85 | negative | negative |
|  | 3 | male | 63 | >85 | negative | negative |
|  | 4 | male | 58 | >90 | negative | negative |
|  | 5 | female | 61 | >80 | negative | negative |
|  | 6 | male | 63 | >80 | negative | negative |
| **Control** |  |  |  |  |  |  |
|  | 1 | male | 60 | negative | negative | negative |
|  | 2 | female | 57 | negative | negative | negative |
|  | 3 | male | 63 | negative | negative | negative |
|  | 4 | female | 66 | negative | negative | negative |
|  | 5 | male | 58 | negative | negative | negative |
|  | 6 | male | 56 | negative | negative | negative |
