# Supplementary figures and images for: Role of long non-coding RNA-RNCR3 in atherosclerosis-related vascular dysfunction
Source: Cell Death Dis. 2016 Jun 2;7(6):e2248–. doi: 10.1038/cddis.2016.145 (PMC5143375; doi:10.1038/cddis.2016.145)

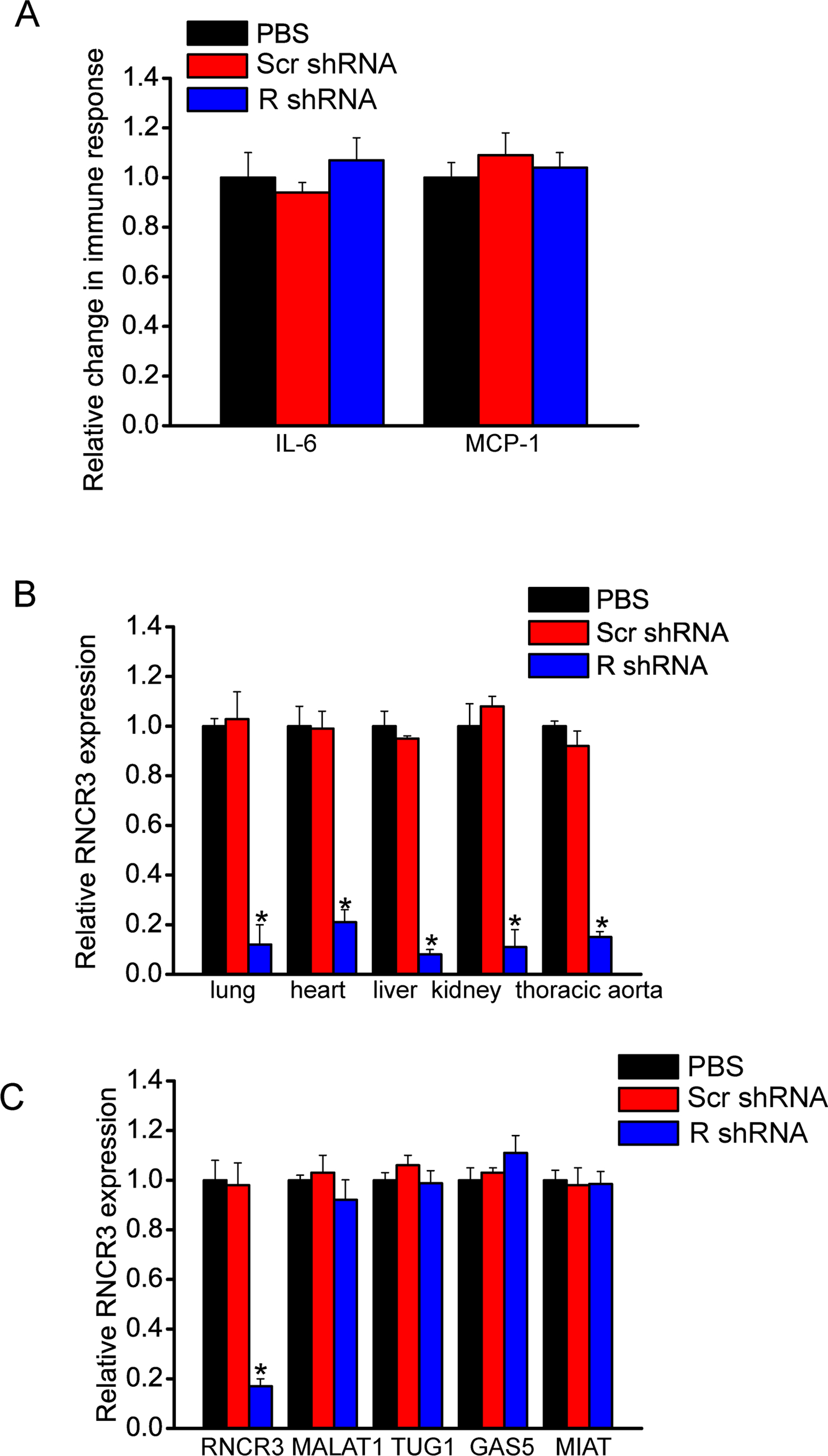

Supplement: Supplementary Figure 1 [file cddis2016145x3.tif]

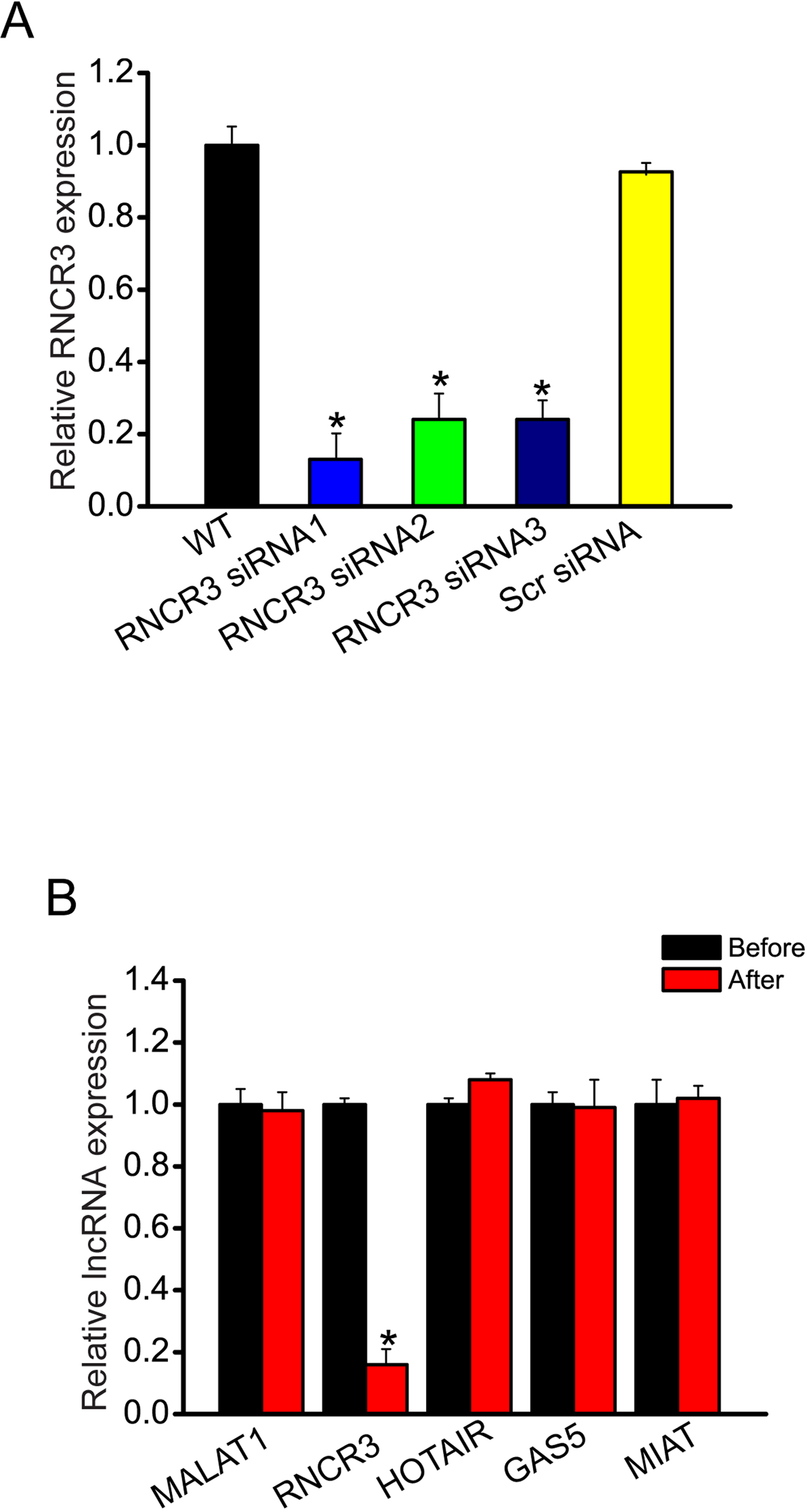

Supplement: Supplementary Figure 2 [file cddis2016145x4.tif]

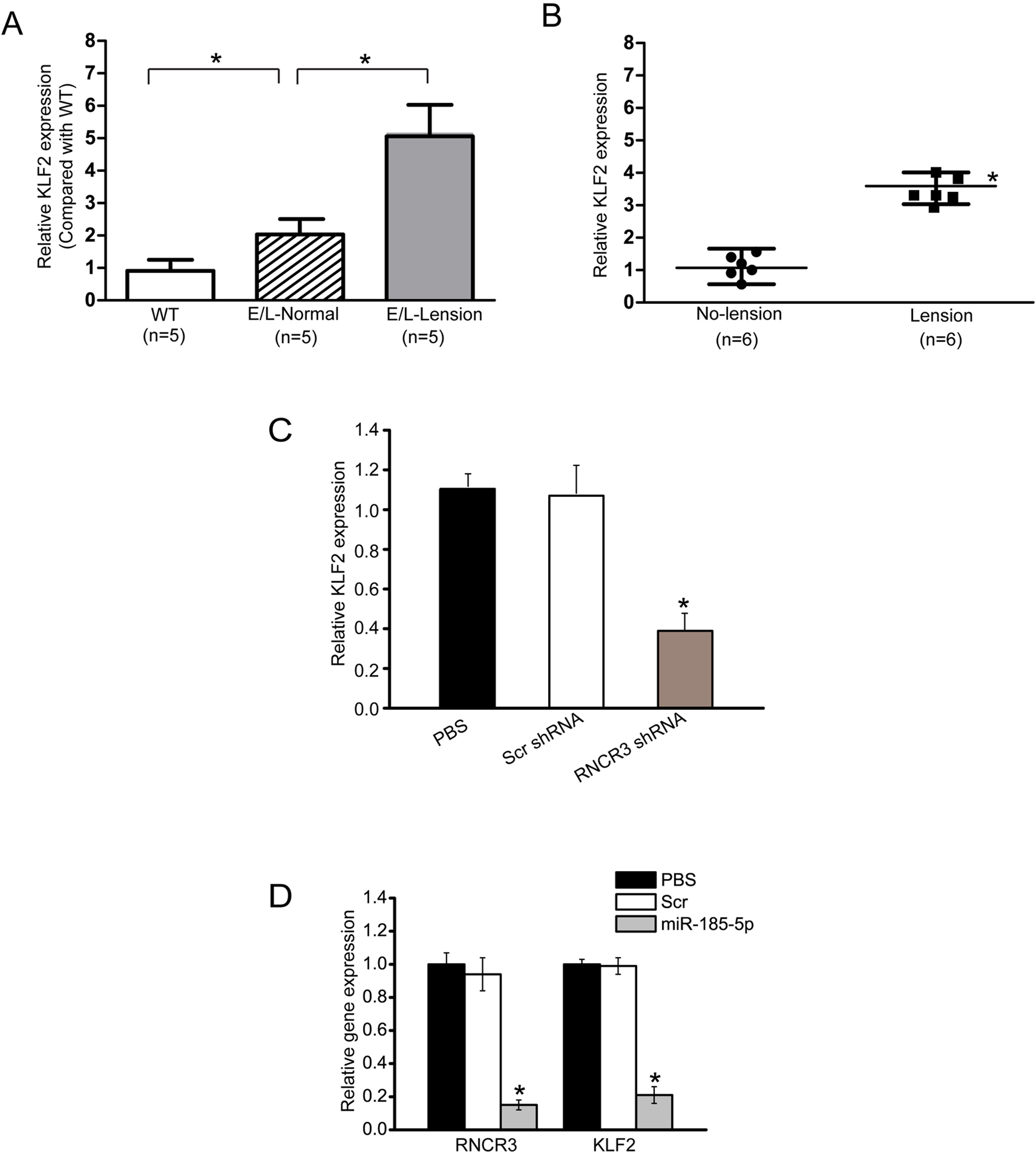

Supplement: Supplementary Figure 3 [file cddis2016145x5.tif]
